# A comprehensive *in silico* analysis for identification of therapeutic epitopes in HPV16, 18, 31 and 45 oncoproteins

**DOI:** 10.1371/journal.pone.0205933

**Published:** 2018-10-24

**Authors:** Heidar Ali Panahi, Azam Bolhassani, Gholamreza Javadi, Zahra Noormohammadi

**Affiliations:** 1 Department of Biology, School of Basic Sciences, Science and Research Branch, Islamic Azad University, Tehran, Iran; 2 Department of Hepatitis and AIDS, Pasteur Institute of Iran, Tehran, Iran; Penn State University School of Medicine, UNITED STATES

## Abstract

Human papillomaviruses (HPVs) are a group of circular double-stranded DNA viruses, showing severe tropism to mucosal tissues. A subset of HPVs, especially HPV16 and 18, are the primary etiological cause for several epithelial cell malignancies, causing about 5.2% of all cancers worldwide. Due to the high prevalence and mortality, HPV-associated cancers have remained as a significant health problem in human society, making an urgent need to develop an effective therapeutic vaccine against them. Achieving this goal is primarily dependent on the identification of efficient tumor-associated epitopes, inducing a robust cell-mediated immune response. Previous information has shown that E5, E6, and E7 early proteins are responsible for the induction and maintenance of HPV-associated cancers. Therefore, the prediction of major histocompatibility complex (MHC) class I T cell epitopes of HPV16, 18, 31 and 45 oncoproteins was targeted in this study. For this purpose, a two-step plan was designed to identify the most probable CD8+ T cell epitopes. In the first step, MHC-I and II binding, MHC-I processing, MHC-I population coverage and MHC-I immunogenicity prediction analyses, and in the second step, MHC-I and II protein-peptide docking, epitope conservation, and cross-reactivity with host antigens’ analyses were carried out successively by different tools. Finally, we introduced five probable CD8+ T cell epitopes for each oncoprotein of the HPV genotypes (60 epitopes in total), which obtained better scores by an integrated approach. These predicted epitopes are valuable candidates for *in vitro* or *in vivo* therapeutic vaccine studies against the HPV-associated cancers. Additionally, this two-step plan that each step includes several analyses to find appropriate epitopes provides a rational basis for DNA- or peptide-based vaccine development.

## Introduction

HPVs are a large branch of the *Papillomaviridae* family, grouped in different genera (Alpha-, Nu-/Mu-, Beta- and Gamma-papillomaviruses), with more than 200 genotypes [1–4]. The classification of Papillomaviruses (PVs) has been based on L1 gene sequence. They are clinically divided into two groups: low-risk HPVs, like HPV 6 and 11, which cause benign lesions (warts and benign papillomas), and high-risk HPVs (hrHPVs), like HPV16 and 18, which are carcinogenic to humans [5–7]. The global ratio of all the malignant diseases attributable to HPV infection is estimated to be 5.2% [8–10]. Almost all the cervical carcinomas and a significant part of anogenital and oropharyngeal malignancies are associated with HPV infections [11].

Currently, It is proven that all the oncogenic HPVs are genetically related, Although, they vary greatly in the prevalence and risk of triggering malignant lesions [12, 13]. According to the International Agency for Research on Cancer evaluation (IARC), twelve HPV types (16, 18, 31, 33, 35, 39, 45, 51, 52, 56, 58, and 59) are known as hrHPV. All hrHPVs belong to the alpha genus in *Papillomaviridae* family. Oncogenicity of some types that classified as probably carcinogenic (HPV 68) or possible carcinogenic (HPV 34, 73, 26, 69, 82, 30, 53, 66, 70, 85, 97, 67, 5 and 8) is still needed to be clarified [14].

The relatively simple genome of HPV contains three regions: the upstream regulatory region (URR), the early region, and the late region. The early and late regions encode six early genes (E1, E2, E4, E5, E6, and E7) and two late genes (L1 and L2), respectively. Among these early proteins, E5, E6, and E7 play a pivotal role in the cell transformation. They can interfere in several cell cycle pathways, especially the alteration of EGFR signaling pathways [15, 16], degradation of p53 [17] and degradation of pRB [18], respectively. These effects result in triggering several cascade events, which cause cell transformation, immune evasion and cancer progression [6, 19–26]. E6 and E7 oncoproteins are known as Ideal targets for the immunotherapy of HPV-associated cancers [27–31] since they are consistently expressed in almost all cervical cancer cells, but not in healthy cells, and are essential for the generation and maintenance of malignancy. Additionally, E5, E6, and E7 oncoproteins are structurally different from human cell proteome. Therefore, their side effects on healthy tissues are expected to be negligible [8].

Currently, there are three commercially available HPV prophylactic vaccines [32]. However, none of them showed an effective therapeutic effect on pre-existing HPV infection or its associated cancers [33–35]. Due to the high prevalence and mortality, there is an urgent need to develop an effective therapeutic HPV vaccine for clearance of these infections/cancers. So far, different therapeutic vaccines have been developed [27–31, 36–45]. However, they have induced inadequate immune responses, and thus further studies are needed to develop an effective therapeutic vaccine.

Among various therapeutic vaccines, peptide-based vaccines have appeared as attractive candidates to treat cervical and other HPV-associated cancers. Peptide-based vaccines have some advantages such as easy production and transportation, high selectivity, multivalency capability and epitope accessibility. With the development of genome sequencing techniques, the prediction of potential B and T cell epitopes has opened a promising view to developing peptide-based vaccines against infectious diseases and cancers. Currently, several therapeutic peptide-based HPV vaccines are in different phases of clinical trials [46].

Host genetic polymorphisms influence the immune response to a pathogen in the target population. HLA genes are the most polymorphic genes in the human genome. The vast HLA polymorphism and restriction phenomenon, result in serious problems in vaccine design and population coverage [47–50] because each allele binds to a particular group of peptides. However, many of HLA-I alleles can be classified by their similar peptide-binding properties into groups, covering over 80% of HLA-A and B alleles. Each HLA-I supertype (HLA-A*01:01, HLA-A*02:01, HLA-A*03:01, HLA-A*24:02, HLA-A*26:01, HLA-B*07:02, HLA-B*08:01, HLA-B*15:01, HLA-B*27:05, HLA-B*39:01, HLA-B*40:01 and HLA-B*58:01) represent a group of HLA molecules which bind to a similar set of peptides [51].

The previous studies have shown that the presence of high immunogenic CD8+ cytotoxic T lymphocytes (CTLs) epitopes in vaccine formulation is essential for inducing a robust immune response. However, the addition of CD4+ T cell epitopes can significantly augment its strength and duration [49, 52, 53]. CD8+ CTLs commonly recognize intracellular-originated peptides presented by MHC-I molecules. They accommodate peptides with 8–11 residues; the ideal length is 9 residues. While CD4+ Helper T Lymphocytes (HTLs) commonly recognize extracellular-originated peptides presented by MHC-II molecules. They accommodate peptides with 10–30 residues or even more; the ideal length is 12–16. The strength of the interaction between a T cell receptor and a peptide-MHC complex (pMHC), depends on the presented peptide and the MHC structure [49, 54]. The binding of a peptide to MHC-I molecule is the most selective stage in the way of peptide presentation [55].

Bioinformatics tools can predict the potential immunogenic epitopes from thousands of epitopes in a short time [56]. Generally, the algorithms of these tools range from ones programmed to determine peptide- MHC molecule binding data to those based on structural similarity, molecular modeling, and molecular docking [57]. Peptides that bind to a specific MHC molecule have sequence similarity. Therefore, peptide sequence patterns have been used to predict their binding to MHC molecules [58]. In recent years, the accuracy of these methods has increased strikingly, and more than 90% of natural epitopes have been recognized at a high specificity of 98% [59]. This improvement in performance was achieved by the expanding experimental binding data, available in the *immune epitope database* (IEDB) *and analysis resource* (http://www.iedb.org/), and by the improvement of machine-learning algorithms [60].

**Fig 1:**
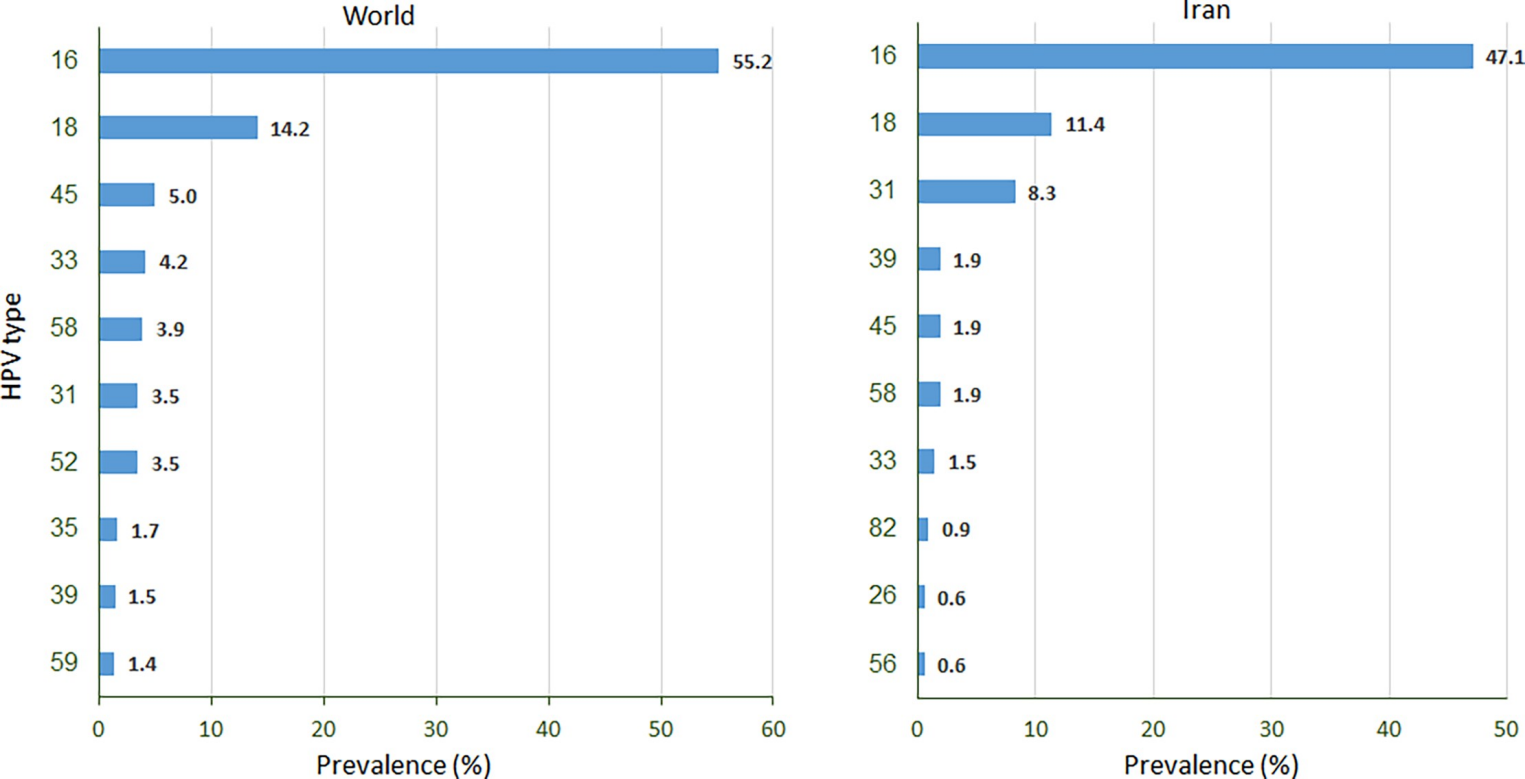
The most prevalent oncogenic HPV types among women with cervical cancer in the world and Iran, 2017 (http://www.hpvcentre.net/datastatistics.php).

Regarding the fundamental importance of epitope prediction in vaccine development, we investigated the best potential CD8+ T cell epitopes from the E5, E6, and E7 oncoproteins of four prevalent hrHPV genotypes (16, 18, 31 and 45) in the world and Iran [61], as shown in Fig 1.

## Materials and methods

### Plan of the study

**Fig 2:**
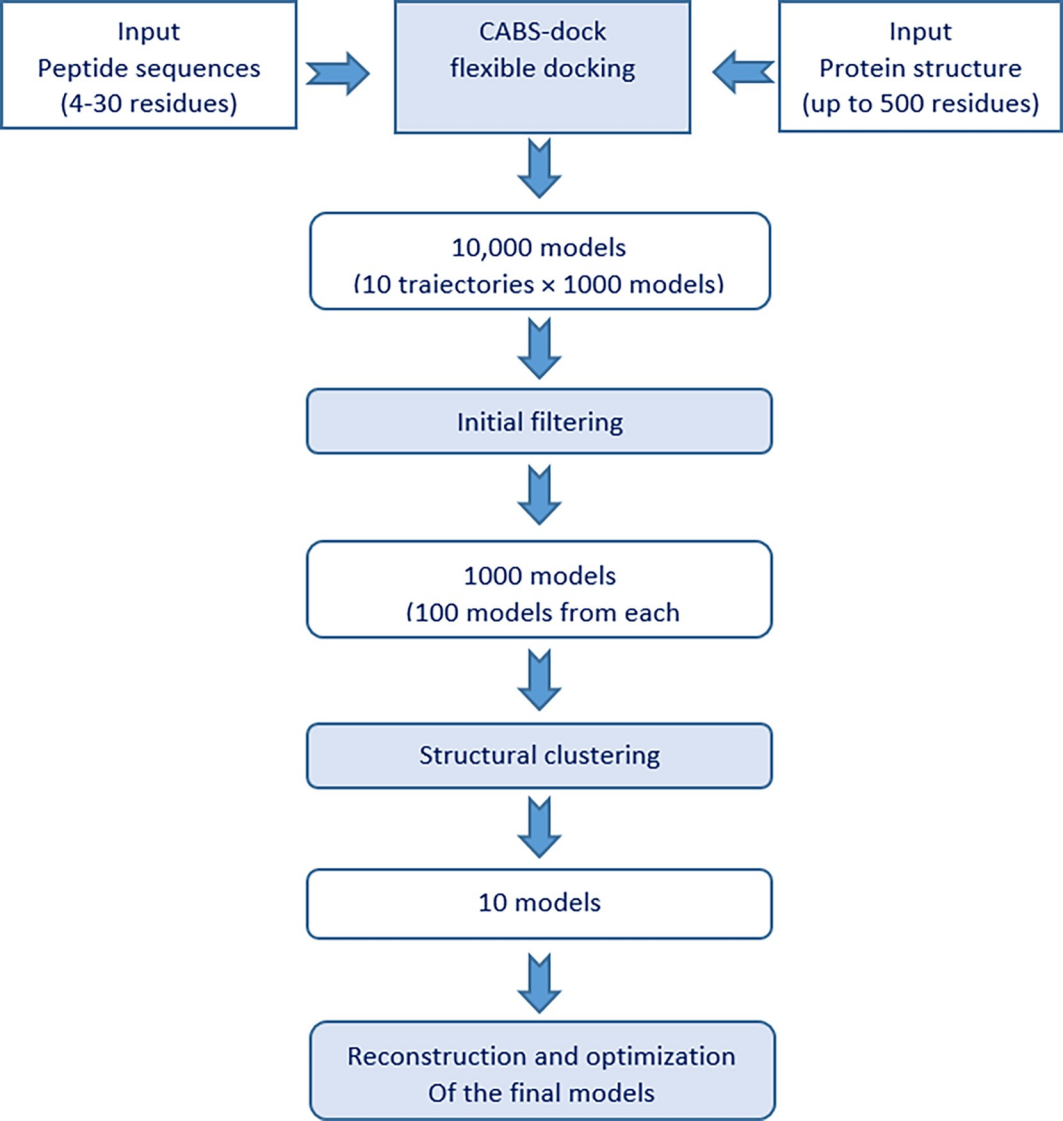
**The flow chart of the study**: It represents the two-step epitope selection plan implemented to identify the most probable epitopes of hrHPV oncoproteins.

A two-step plan was designed to identify the most probable CD8+ T cell epitopes (Fig 2). For the first step, MHC-I and II binding, MHC-I processing, MHC-I population coverage and MHC-I immunogenicity prediction analyses, and for the second step, MHC-I and II protein-peptide docking, epitope conservation, and cross-reactivity with host antigens analyses were considered. The second step analyses were performed only for the selected peptides in the first step.

### Protein sequences

In Jan 2018, in order of priority, the RefSeq, reviewed or unreviewed sequences of hrHPV oncoproteins (E5, E6, and E7) were retrieved from the National Center for Biotechnology Information database (NCBI) (http://www.ncbi.nlm.nih.gov/) and UniProtKB/Swiss-Prot database (http://www.uniprot.org/). The isoform sequences of HPV16, 18, 31, and 45 oncoproteins were retrieved from HPV T cell Antigen Database (http://cvc.dfci.harvard.edu/hpv/HTML/search.php). All the sequences are accessible in supporting information (S1 File).

### MHC-I binding prediction

Binding of epitopes to MHC-I molecules is an essential step for antigen presentation to CTLs. Herein, it was predicted by four online servers, as illustrated in Table 1. The HLA supertypes and frequently occurring HLA-I alleles provided by the servers were included in the analysis. However, when an allele (e.g., HLA-B*14:02) was not provided, but its allele group (i.e., HLA-B*14) was available, we used the allele group instead of the allele. The used human and mouse alleles, or allele groups are provided in supporting information (S1 Table).

**Table 1:** Predictor servers used for MHC-I epitope binding prediction.

Server name	Link	Prediction method	References
IEDB MHC-I binding (IEDB recommended)	http://tools.iedb.org/mhci/	ANN, SMM and CombLib_Sidney2008	[60, 62–65]
NetMHCpan4	http://www.cbs.dtu.dk/services/NetMHCpan/	ANN	[66]
Rankpep	http://imed.med.ucm.es/Tools/rankpep.html	PSSM	[67, 68]
SYFPEITHI	http://www.syfpeithi.de/0-Home.htm	Published motifs	[54]

### IEDB MHC-I binding prediction

Currently, eight prediction methods are available in the IEDB MHC-I binding prediction tool, *i*.*e*., *IEDB recommended* [62], *Consensus* [69], *NetMHCpan3* [59, 70], *artificial neural network* (*ANN)* [71, 72], *SMM with a peptide-MHC binding energy covariance matrix (SMMPMBEC*) [73], *stabilized matrix method (SMM*) [74], *CombLib_Sidney2008* [75], *PickPocket* [76], *netMHCcons* [77] and *netMHCstabpan* [78]. The *IEDB-recommended* and *consensus* are not Independent methods; they use *ANN*, *SMM* and *CombLib_Sidney2008* methods to generate a representative index for each predicted pMHC; The median of percentile ranks (PRs) or binding scores obtained from the used methods is reported as a representative PR or *consensus score* in the *IEDB-recommended* or *consensus* method respectively. The PR is calculated by comparing the *half maximal inhibitory concentration* (IC_50_) of subjected peptide against a group of random peptides from Swiss-Prot database. The IC_50_ value, expressed as nanomolar, shows binding affinity. The lower IC_50_ or PR means higher binding affinity. As a rough guideline, peptides with IC_50_ values <50_nM_ are considered as high affinity, 50-500_nM_ intermediate affinity and more than 500-5000_nM_ low affinity. No known T cell epitope has got an IC_50_ value >5000_nM_ to date [60].

In this study, *IEDB recommended* method was used. The outputs for each pMHC in this method consisted of a median PR, a method-specific IC_50_, and a method-specific PR. Predictions were made against 76 frequently occurring human MHC-I alleles (including 12 HLA supertypes) and 6 MHC-I mouse alleles. Epitope length was set on 8, 9, 10, and 11mer. Peptides with median PR <2.0 are applied for the analysis.

#### NetMHCpan4 MHC-I binding prediction

NetMHCpan4 server predicts binding of peptides to the known MHC molecules using ANNs method. It is trained on a combination of naturally eluted ligands (55 human and mouse MHC-I alleles) and binding affinity data (172 MHC molecules from human, mouse, cattle, primates, and swine). Besides, the user can perform a prediction against any custom MHC-I molecule by uploading its full-length sequence [66].

In this study, predictions were performed for 8, 9, 10, and 11mer peptides against 76 frequently occurring human MHC-I alleles and 8 MHC-I mouse alleles. PR thresholds for strong and weak binders were set on 0.5 and 2.0, respectively. Peptides with PR <2.0 were applied for the analysis.

#### Rankpep MHC-I binding prediction

Rankpep predicts binder peptides of a given protein sequence or sequence alignments to MHC-I and II molecules. The algorithm of Rankpep based on the comparison of sequence similarities, using *position-specific scoring matrices* (PSSMs) method. It employs profiles of a group of aligned peptides recognized to bind to a specific MHC molecule and creates a consensus sequence by determining the most common residue for each position. Then, it allocates an optimal score to the consensus sequence, compares the score of the subjected peptide with the optimal score, and gives the peptide a percentile optimal value for comparison. Finally, it highlights strong binders in red [67, 68].

Herein, the prediction was made against 31 frequently occurring HLA-I and 7 H2-I alleles. The server did not provide all common lengths of epitopes for all the MHC alleles. Thus, the used alleles and their provided epitope lengths are shown together, as given in supporting information (S1 Table).

#### SYFPEITHI MHC-I binding prediction

SYFPEITHI (http://www.syfpeithi.de/0-Home.htm/) is a database of over 7000 published and verified peptide sequences of human, mouse, and other organisms, known as natural binders of MHC-I and II molecules. When SYFPEITHI analyzes a peptide for binding prediction against a specific MHC-I allele, its scoring system evaluates every residue of the query and gives it an arbitrary value between 1 and 15, according to whether it is an anchor, auxiliary anchor, or preferred residue. It allocates the value 1 to those residues which slightly preferred in that particular position, 15 to the Ideal anchor residues, and -1 to -3 to those residues which exhibit an adverse effect on the binding ability. The sum of these values is the score of the peptide. The maximal score could vary between different MHC alleles [54, 79].

Herein, the prediction was made against 26 frequently occurring HLA-I alleles and 5 H2-I alleles. Epitope length was set on 8, 9, 10, and 11mer. Every predicted pMHC which got a score less than 70% of the reference sequence score was excluded from the analysis. The allele-specific reference sequence was selected from Rankpep's *consensus sequence* [68], or our SYFPEITHI predicted epitopes, whichever got the highest score in SYFPEIHI server. The reference sequences, their sources, and their scores are given in supporting information (S2 Table).

### MHC-II binding prediction

Recognition of high immunogenic CD8+ T cell epitopes was the primary aim of this study. Therefore, all predictions were primarily made against epitopes with 8–11 residue length. However, it was valuable to determine that which 9mer MHC-I epitope is the core peptide of the MHC-II epitope(s) too. The core peptide lies on the MHC-II molecule grooves, and play the central role in constructing pMHC. With this strategy, the short minimal predicted epitopes could be used in designing of synthetic long peptides (SLPs), resulting in peptide loading to both MHC-I and II molecules.

#### IEDB MHC-II binding prediction

In this study, the MHC-II binding prediction was made by IEDB MHC-II binding predictor (http://tools.iedb.org/mhcii/) [60, 63, 64]. IEDB possess seven prediction methods for MHC-II binding prediction: *IEDB-recommended*, *consensus* [63], *NetMHCIIpan*[80], *NN- align* [81], *SMM-align* [82], *Combinatorial Libraries* [75] and *Sturniolo's method* [83]. Herein, the *IEDB-recommended* method was used, and all peptides with PR<2.0 were selected for the analysis.

The prediction was made against 35 human alleles (IEDB reference set) and three mouse alleles, given in supporting information (S3 Table). The server has fundamentally set the epitope length on 15mer. Each *IEDB-recommended* method participated in the prediction process offered a core Peptide (9mer) for each predicted epitope (15mer). We associated the 9mer MHC-II core peptides with the 9mer MHC-I predicted epitopes to determine that which MHC-I epitope is the core peptide of the MHC-II epitope(s) too.

### MHC-I processing prediction

MHC-I T cell epitope processing predictions of E5, E6, and E7 oncoproteins are made by the *IEDB combined predictor* (http://tools.iedb.org/processing/). This tool combines predictors of three main steps of MHC-I antigen presentation pathway (proteasomal processing, transporter associated with antigen processing (TAP) transport, and MHC-I binding) and calculates a total processing score for each predicted epitope. It allows the user to choose a method from *ANN*, *SMM*, *SMMPMBEC*, *Comblib_Sidney2008*, *NetMHCpan*, *NetMHCcons* and *PickPocket* methods for the binding prediction. In the current update (2018), the IEDB team has changed the choice of the recommended prediction method for the processing tool to be *NetMHCpan 3*.*0* rather than a *consensus*, since the processing tools requiring an IC_50_ value, which the *consensus* method does not provide. Furthermore, *NetMHCpan 3*.*0* has provided all MHC alleles and has performed the predictions very well in recent comparisons [65].

There are two types of proteasomes, the housekeeping types which are expressed instinctively, and immuno types which are provoked by IFN-γ secretion. The immunoproteasomes are believed to improve the efficiency of antigen presentation [62, 65]. In this study, the immunoproteasome option was selected.

The program outputs for every predicted epitope consisted of proteasome score, TAP score, MHC score, processing score (proteasome + TAP score), total score (Proteasome + TAP + MHC score), and MHC-I IC_50_. The TAP scoring system calculates a–log (IC_50_) value for the binding of a peptide (or N-terminal of its precursors) to the TAP molecules. The higher TAP score, the higher transport rate. [62, 65, 84].

Herein, the analysis was made against the human and mouse MHC-I alleles used later in the IEDB binding prediction, with the *IEDB-recommended* method and other default settings of the program. Epitopes with IC_50_ <1000 _nM_ for HLA-I alleles and <5000 _nM_ for H2-I alleles were included in the analysis.

### MHC-I immunogenicity prediction

Several factors could clarify the difference between epitope and non-epitope peptides; An essential factor is epitope immunogenicity, *i*.*e*., it could be recognized by T cells. Some amino acids, particularly those with large and aromatic side chains (especially tryptophan, phenylalanine, and Isoleucine), are associated with immunogenicity. Moreover, the positions P4–6 of a peptide are more critical for immunogenicity [85].

In this study, the MHC-I immunogenicity of all predicted epitopes was determined by the IEDB web server (http://tools.iedb.org/immunogenicity/)[85]. This tool uses the properties of amino acids and their locations to predict the immunogenicity of a pMHC. The default option was selected to specify which positions of the query peptide to be masked from the analysis, because it masked positions which are also suggested for the most frequent human MHC-I allele, HLA-A*02:01.

### Population coverage prediction

IEDB population coverage prediction tool (http://tools.iedb.org/population/) [86] is used to predict the HLA-I population coverage of all 8-11mer predicted epitopes in the first step. This tool can accept a target population by two query levels: 1) area-country-ethnicity and 2) ethnicity alone. It can integrate allele frequency information retrieved from the *Allele Frequency Net Database* (AFND) (http://www.allelefrequencies.net/default.asp) [87]. IEDB also accepts custom populations with allele frequencies defined by users. Since, HLA-I and HLA-II T cell epitopes elicit immune responses from two different T cell populations (CTL and HTL, respectively), the server provided three different population coverage modes: 1) HLA-I lonely, 2) HLA-II lonely, and 3) HLA-I and HLA-II together.

Herein, the MHC-I promiscuous predicted epitopes and their binding HLA-I alleles (IC_50_<500_nM_ or PR<2.0) were entered as inputs for the analysis against the world population.

### Molecular docking analysis

**Fig 3:**
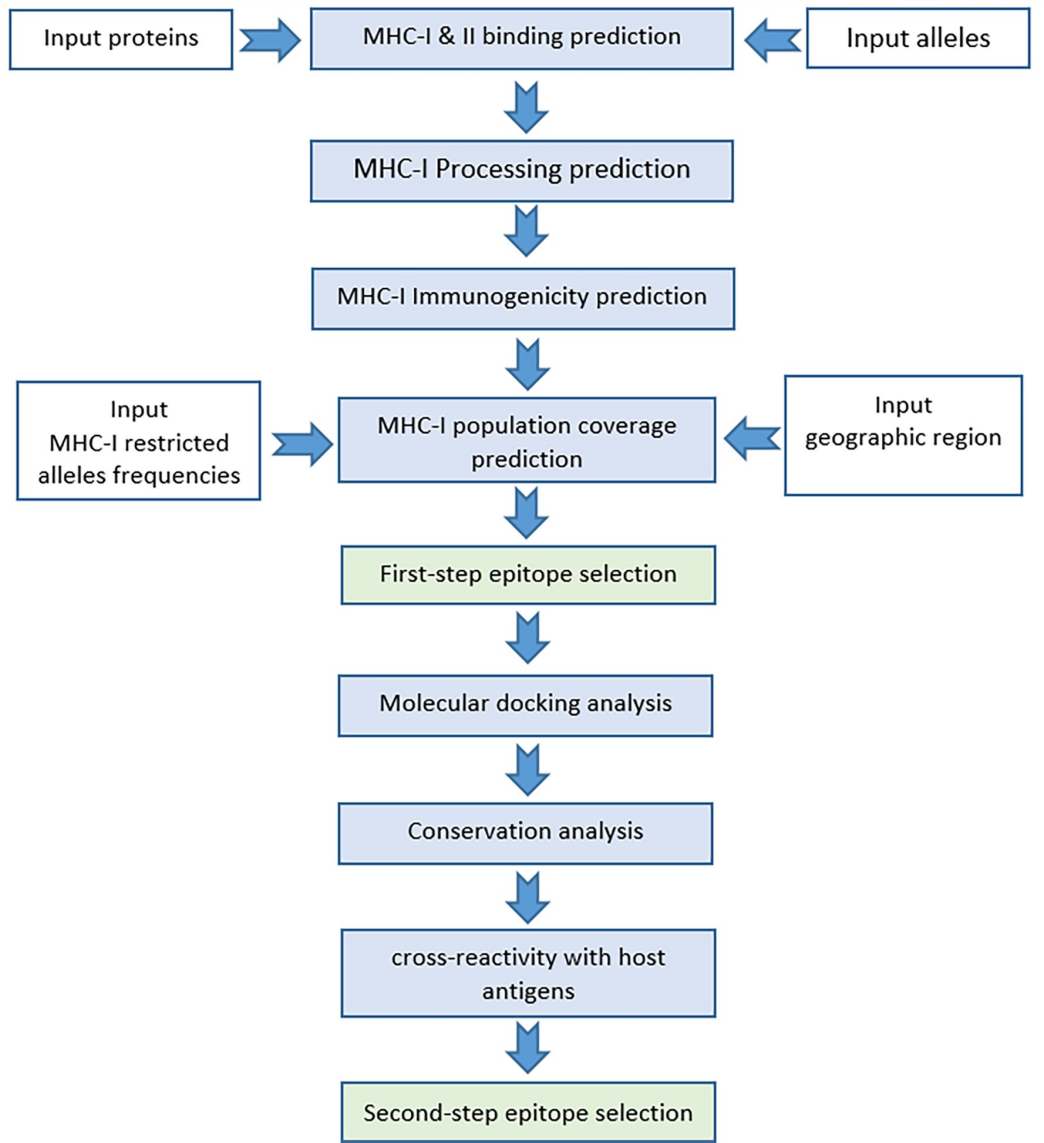
**The pipeline of CABS-dock protocol:** The fully automated CABS-dock procedure contains four main stages, shown in the blue boxes.

The primary aim of molecular docking is the prediction of the binding site of a ligand at a protein receptor surface, and then docking and modeling the ligand into the recognized site. In this study, the binding ability of the first step selected peptides to human and mouse MHC molecules, was analyzed by CABS-dock (http://biocomp.chem.uw.edu.pl/CABSdock/) server. The server uses a multistage procedure that involves multiple programs, with the Cα–Cβ–side chain (CABS) model at its heart. The detailed information about these stages is given in supporting information (S2 File) [88, 89]. Also, Fig 3 shows the pipeline of CABS-dock protocol [88].

CABS-dock gets the 3D structure of the receptor and the sequence of the peptide as obligatory inputs. Furthermore, there are some non-obligatory inputs as recommendations which could improve outputs. In this study, duplicate dockings for each peptide (6240 dockings in total) were done against the most significant human/mouse MHC-I and II molecules which had at least one well-structured protein data bank (PDB) file in the RCSB Protein Data Bank (https://www.rcsb.org/), as shown in Table 2. These PDB files are in the complex with their peptidic ligand and some X-ray crystallography solution molecules (heteroatoms). Thus, these excess molecules, as well as redundant MHC molecules were removed before executing docking process. Since, the binding site of epitopes on the MHC molecules was well-known previously, the unlikely regions to bind masked before the analysis.

**Table 2:** MHC alleles used for molecular docking analysis against the selected peptides in the first step.

Human MHC-I		Human MHC-II		Mouse MHC-I		Mouse MHC-II	
Allele	PDB ID	Allele	PDB ID	Allele	PDB ID	Allele	PDB ID
HLA-A*02:01	4UQ3	HLA-DRB1*01:01	4AH2	H-2-Db	1JUF	H-2-IAb	4P23
HLA-A*24:02	5HGA	HLA-DRB1*03:01	2Q6W	H-2-Dd	5IVX	H-2-IAd	2IAD
HLA-A*01:01	4NQV	HLA-DRB1*04:01	5LAX	H-2-Kb	4PV9	H-2-Ag7	1ESO
HLA-A*03:01	3RL2	HLA-DRB1*11:01	6CPL	H-2-Kd	5GSV	H-2-IEk	1FNG
HLA-A*11:01	1X7Q	HLA-DRB1*15:01	5V4M	H-2-Kk	1ZTV		
HLA-B*07:02	5EO1	HLA-DRB5*01:01	1FV1	H-2-Ld	1LDP		
HLA-B*08:01	3SPV						
HLA-B*27:05	1OGT						
HLA-B*35:01	3LKN						
HLA-B*27:05	4UQ3						

CABS-dock returns ten representative models (medoids) as the best-simulated models and ranks them by cluster density (CD). Cluster density is equal to the number of elements in a cluster divided by their average ligand *root mean square deviation* (RMSD). The higher CD value implies greater accuracy. Ligand RMSD value shows the differentiation measure between cluster elements. As a guideline; RMSD < 3.0 Å means high accuracy; RMSD ≥ 3.0 and ≤ 5.5 Å means medium accuracy and RMSD > 5.5 Å means low accuracy [88]. Herein, the RMSD and CD of the best-simulated models were selected for the analysis. The best model, which has the highest CD value, is not necessarily the top-ranked model, because, in some cases, peptides were not attached to their binding site properly. Thus, these malformed models were excluded from the analysis. It is important to note that, due to the different frequency of MHC alleles in human populations, the equal CD value of different MHC alleles, don’t have equal value regarding population coverage. Thus, to involve the effect of population coverage, the CD value of every model was multiplied by its allele population coverage (divided by hundreds for more facility) to obtain a weighted index. Then, the sum of all HLA-I or II weighted indexes of each peptide was calculated to get a total docking score (TDS), used as a score to compare the candidate peptides. It is the first time that the TDS has been formulated and used for this purpose. This formula is also applicable to the similar docking scores obtained from other servers.

### Epitope conservancy analysis

The use of highly conserved epitopes in a vaccine formulation reduces the risk of tumor immune escape and provides broader protection against different virus strains or genotypes. Thus, the conserved areas are preferred to use in therapeutic vaccines, if they are appropriate epitopes. Herein, the epitope conservancy analyses for the first step selected peptides were done in three levels:

1. Inter-isoform conservancy: the percent of conservancy between all isoforms of each E5, E6, or E7 oncoprotein.
2. Inter-type conservancy: the percent of conservancy between HPV16 and 31 (alpha-Papillomavirus 9), as well as between HPV18 and 45 (alpha- Papillomavirus 7).
3. Inter-hrHPV conservancy: complete (100%) conservancy between all hrHPVs (HPV16, 18, 31, 33, 35, 39, 45, 51, 52, 56, 58, and 59).

The selected peptides in the first step were analyzed to find inter-strains and inter-types conservancy percentages by IEDB tool, *conservation across antigens*, (http://tools.iedb.org/conservancy/). The inter-hrHPVs conservancy analysis was done by the IEDB and ExPASy ClustalW servers (https://embnet.vital-it.ch/software/ClustalW.html).

### Cross-reactivity with host antigens

Cross-reactivity with host antigens can cause adverse immune responses. Therefore, the selected peptides in the first step were checked for similarities with the mouse and human proteomes by the NCBI BLASTp tool (https://blast.ncbi.nlm.nih.gov/Blast.cgi).

## Results

Regarding the studies, different peptides usually get different scores/ranks in different analyses. This inconsistency indicates that these results needed to be analyzed with an integrated approach. Indeed, integrated approach is more practical and efficient in such conditions in comparison with *analysis by analysis filtering* approach, in which those epitopes are chosen for the next analysis that have gotten an acceptable score in the previous analysis. Herein, the integrated approach was applied in both steps of epitope selection.

Since the ultimate goal of the discovery of therapeutic epitopes is to use them in human vaccines, only the scores/ranks of human alleles were used to rank epitopes in some studies. However, investigators usually test therapeutic vaccines on mouse species in preclinical trials, thus in the current study, the binding status of the predicted epitopes to mouse MHC-I alleles was also studied by several binding predictors and molecular docking, as well.

As stated above, CTL-mediated responses play a crucial role in killing the malignant cells. Besides, the binding of epitopes to MHC-I molecules is the most selective step for antigen presentation to CTLs. Therefore, in the first step, the selection was made primarily by the comparison of obtained MHC-I binding, processing and immunogenicity scores/ranks, and population coverage percentages. However, the MHC-II binding ranks were actually of secondary importance to the selection process as an added advantage. Additionally, the population coverage has a dual application. First, it determines the coverage of a given peptide in the target population. Second, it is the best index for summarizing and evaluating of the HLA-I binding predictions too, since it is calculated from the results of HLA-I binding prediction analyses.

In the first step, ten peptides (Tables 3–5) from each HPV genotype oncoprotein (120 peptides in total), which got better results in the first step analyses were selected for the second step analyses, including protein-peptide molecular docking, epitope conservation, and cross-reactivity with host antigens. The individual detailed results of the MHC-I and II binding (S3 File), MHC-I immunogenicity (S4 File) and MHC-I population coverage (S5 File) predictions, as well as, MHC-I and II molecular docking (S6 File) and epitope conservation (S7 File) analyses are given in supporting information, as 15 Excel files. Indeed, CABS-dock returns ten representative medoids as the best-simulated models and ranks them by cluster density (CD). Cluster density is derived from two factors (the number of elements in a cluster and their average ligand RMSD) that is an advantage for this server.

**Table 3:** The predicted epitopes from E5 oncoproteins in the first step selection.

Protein	Epitope	Location	Immunogenicity	Population	Proteasomal	TAP	Processing
		(length)	Score	Coverage	Cleavage score	score	score
HPV16-E5	FLIHTHARF	72–80 (9)	0.21	97.75%	1.31	1.12	2.43
	SAFRCFIVY	55–63 (9)	0.28	70.69%	1.55	1.43	2.98
	STYTSLIIL	37–45 (9)	0.06	99.70%	1.67	0.53	2.20
	YTSLIILVL	39–47 (9)	0.20	99.93%	1.57	0.40	1.97
	FLLCFCVLL	15–23 (9)	0.03	62.23%	1.47	0.39	1.86
	YIIFVYIPL	63–71 (9)	0.30	76.67%	1.18	0.51	1.70
	LSVSTYTSL	34–42 (9)	-0.18	93.47%	1.52	0.49	2.01
	FVYIPLFLI	66–74 (9)	0.20	89.59%	1.14	0.34	1.48
	FIVYIIFVY	60–68 (9)	0.39	53.36%	1.36	1.32	2.68
	IIFVYIPLF	64–72 (9)	0.19	99.99%	1.28	1.19	2.47
HPV18-E5	ATAFTVYVF	47–55 (9)	0.23	100.0%	1.50	1.10	2.59
	CAYAWVLVF	29–37 (9)	0.31	78.10%	1.45	1.20	2.65
	SPATAFTVY	45–53 (9)	0.26	82.84%	1.46	1.17	2.63
	MCAYAWVLVF	28–37 (10)	0.32	96.23%	1.45	1.13	2.58
	YAWVLVFVY	31–39 (9)	0.26	62.06%	1.29	1.43	2.71
	LHIHAILSL	64–72 (9)	0.13	84.77%	1.51	0.47	1.98
	FTVYVFCFL	50–58 (9)	0.18	79.20%	1.36	0.47	1.83
	LLLHIHAIL	62–70 (9)	0.30	57.57%	1.57	0.45	2.02
	TSPATAFTVY	44–53 (10)	0.28	90.18%	1.46	1.28	2.74
	MLLLHIHAI	61–69 (9)	0.19	85.90%	1.05	0.21	1.25
HPV31-E5	FVIHTHASF	72–80 (9)	0.08	100.0%	1.11	1.17	2.28
	LSVSVYATL	34–42 (9)	-0.06	100.0%	1.48	0.49	1.97
	SVYATLLLL	37–45 (9)	0.05	99.80%	1.28	0.60	1.88
	VVFIYIPLF	64–72 (9)	0.28	99.59%	1.41	1.21	2.62
	VSVYATLLL	36–44 (9)	0.07	100.0%	1.43	0.52	1.95
	VYATLLLLI	38–46 (9)	0.02	99.96%	1.29	0.40	1.70
	YVVFIYIPL	63–71 (9)	0.36	77.99%	1.13	0.51	1.64
	LIHTHARFL	73–81 (9)	0.05	100.0%	1.50	0.47	1.97
	FLLCFCVLL	15–23 (9)	0.03	57.05%	1.47	0.39	1.86
	FIYIPLFVI	66–74 (9)	0.23	70.90%	1.35	0.35	1.70
HPV45-E5	CAFAWLLVF	29–37 (9)	0.30	69.65%	1.35	1.17	2.53
	YVCAFAWLL	27–35 (9)	0.35	52.04%	1.45	0.49	1.94
	VYVCAFAWLL	26–35 (10)	0.33	99.98%	1.45	0.62	2.06
	LHMHALHTL	64–72 (9)	0.05	99.56%	1.58	0.47	2.05
	VITSPLTAF	42–50 (9)	-0.12	98.75%	1.41	1.19	2.59
	FLLCFSVCL	6–14 (9)	-0.10	56.13%	1.68	0.41	2.09
	FAWLLVFLF	31–39 (9)	0.18	59.90%	1.12	1.22	2.34
	VYVCAFAWL	26–34 (9)	0.27	99.86%	1.16	0.62	1.77
	SPLTAFAVY	45–53 (9)	0.24	57.26%	1.32	1.17	2.49
	MFVLHMHAL	61–69 (9)	-0.08	92.98%	1.64	0.48	2.12

**Table 4:** The predicted epitopes from E6 oncoproteins in the first step selection.

Protein	Epitope	Location	Immunogenicity	Population	Proteasomal	TAP	Processing
		(length)	Score	Coverage	Cleavage score	score	score
HPV16-E6	FAFRDLCIVY	52–61 (10)	0.19	83.26%	1.94	1.24	3.18
	IVYRDGNPY	59–67 (9)	0.09	86.66%	1.13	1.42	2.55
	CYSLYGTTL	87–95 (9)	0.02	99.94%	1.58	0.49	2.06
	VYDFAFRDL	49–57 (9)	0.33	99.99%	1.35	0.41	1.75
	KFYSKISEY	75–83 (9)	-0.33	99.57%	1.39	1.49	2.88
	SEYRHYCYSL	81–90 (10)	-0.07	99.96%	1.59	0.47	2.25
	KLPQLCTEL	18–26 (9)	-0.09	99.98%	1.41	0.52	1.93
	ISEYRHYCY	80–88 (9)	0.08	24.94%	1.57	1.28	2.85
	EYRHYCYSL	82–90 (9)	-0.11	99.91%	1.59	0.49	2.08
	YCYSLYGTTL	86–95 (10)	-0.10	99.99%	1.58	0.49	2.06
HPV18-E6	KLPDLCTEL	13–21 (9)	0.05	99.99%	1.65	0.44	2.09
	TVLELTEVF	37–45 (9)	0.23	99.27%	1.43	1.20	2.63
	FAFKDLFVVY	47–56 (10)	0.01	86.98%	1.90	1.23	3.12
	LELTEVFEF	39–47 (9)	0.33	49.80%	1.40	1.07	2.47
	DFYSRIREL	70–78 (9)	0.11	87.51%	1.64	0.44	2.08
	FEFAFKDLF	45–53 (9)	0.01	75.51%	1.15	1.07	2.22
	FAFKDLFVV	47–55 (9)	-0.05	96.60%	1.02	0.09	1.11
	AFKDLFVVY	48–56 (9)	0.11	95.40%	1.90	1.36	3.25
	LQDIEITCVY	25–34 (10)	0.38	51.48%	1.31	1.25	2.56
	SVYGDTLEK	84–92 (9)	0.14	58.01%	0.94	0.31	1.24
HPV31-E6	FAFTDLTIVY	45–54 (10)	0.26	93.1%	1.87	1.24	3.11
	RYSVYGTTL	80–88 (9)	0.07	100.0%	1.48	0.61	2.09
	KVSEFRWYRY	72–81 (10)	0.45	68.2%	1.43	1.40	2.83
	FRWYRYSVY	76–84 (9)	0.00	66.2%	1.45	1.32	2.77
	YRYSVYGTTL	79–88 (10)	-0.04	100.0%	1.48	0.55	2.03
	LSSALEIPY	15–23 (9)	0.18	61.3%	1.00	1.21	2.22
	KVSEFRWYR	72–80 (9)	0.40	99.9%	0.90	0.77	1.67
	AFTDLTIVY	46–54 (9)	0.20	97.6%	1.87	1.34	3.21
	KLHELSSAL	11–19 (9)	-0.17	99.96%	1.39	0.54	1.93
	FAFTDLTIV	45–53 (9)	0.20	95.45%	1.03	0.11	1.13
HPV45-E6	IVYRDCIAY	54–62 (9)	0.16	81.72%	1.55	1.42	2.97
	KLPDLCTEL	13–21 (9)	0.05	99.99%	1.65	0.44	2.09
	YSRIRELRY	72–80 (9)	0.32	85.96%	1.46	1.25	2.71
	ATLERTEVY	37–45 (9)	0.29	97.51%	1.40	1.37	2.79
	FAFKDLCIVY	47–56 (10)	-0.08	83.30%	1.90	1.28	3.15
	DFYSRIREL	70–78 (9)	0.11	98.61%	1.56	0.44	1.99
	YQFAFKDL	45–52 (8)	0.01	80.70%	1.44	0.51	1.95
	NPAEKRRHL	113–121 (9)	0.02	45.86%	1.62	0.33	1.95
	RTEVYQFAF	41–49 (9)	0.08	99.99%	1.49	1.06	2.55
	YSRIRELRYY	72–81 (10)	0.33	80.75%	1.30	1.25	2.56

**Table 5:** The predicted epitopes from E7 oncoproteins in the first step selection.

Protein	Epitope	Location	Immunogenicity	Population	Proteasomal	TAP	Processing
		(length)	Score	Coverage	Cleavage score	score	score
HPV16-E7	RAHYNIVTF	49–57 (9)	0.18	99.98%	1.48	1.18	2.66
	STHVDIRTL	71–79 (9)	0.27	99.35%	1.79	0.42	2.21
	LEDLLMGTL	79–87 (9)	-0.13	51.13%	1.70	0.29	1.98
	LQPETTDLY	15–23 (9)	0.18	81.48%	1.16	1.24	2.40
	TLHEYMLDL	7–15 (9)	-0.05	96.26%	1.17	0.37	1.53
	LLMGTLGIV	82–90 (9)	0.11	40.60%	0.97	0.11	1.08
	DRAHYNIVTF	48–57 (10)	0.22	80.89%	1.48	1.00	2.48
	GQAEPDRAHY	43–52 (10)	0.23	49.47%	1.57	1.30	2.87
	QAEPDRAHY	44–52 (9)	0.14	57.01%	1.57	1.22	2.79
	GTLGIVCPI	85–93 (9)	0.15	42.66%	0.76	0.17	0.93
HPV18-E7	SSADDLRAF	78–86 (9)	0.11	99.69%	1.40	1.07	2.47
	FQQLFLNTL	86–94 (9)	0.07	95.89%	1.71	0.46	2.17
	QLFLNTLSF	88–96 (9)	-0.05	88.83%	1.30	1.14	2.44
	TLQDIVLHL	7–15 (9)	0.16	99.98%	1.57	0.47	2.04
	LRAFQQLFL	83–91 (9)	-0.03	62.34%	1.74	0.45	2.20
	RAFQQLFL	84–91 (8)	-0.10	99.98%	1.74	0.62	2.36
	QQLFLNTLSF	87–96 (10)	0.03	39.85%	1.30	1.10	2.40
	RAEPQRHTM	53–61 (9)	0.01	99.95%	0.97	0.26	1.22
	SADDLRAF	79–86 (8)	0.10	74.99%	1.40	1.09	2.49
	RAFQQLFLNTL	84–94 (11)	-0.08	64.77%	1.70	0.62	2.32
HPV31-E7	TSNYNIVTF	49–57 (9)	0.17	99.95%	1.51	1.01	2.51
	TLQDYVLDL	7–15 (9)	0.02	99.77%	1.30	0.34	1.64
	GQAEPDTSNY	43–52 (10)	0.02	44.68%	1.48	1.30	2.78
	QAEPDTSNY	44–52 (9)	-0.06	56.83%	1.48	1.22	2.70
	QPEATDLHCY	16–25 (10)	0.12	42.18%	1.47	1.10	2.57
	TPTLQDYVL	5–13 (9)	-0.07	45.76%	1.63	0.19	1.81
	LLMGSFGIV	82–90 (9)	0.03	46.52%	1.02	0.10	1.12
	YVLDLQPEA	11–19 (9)	-0.05	79.18%	1.42	-0.23	1.18
	IRILQELLM	76–84 (9)	0.00	62.34%	0.96	0.20	1.16
	VDIRILQEL	74–82 (9)	0.18	27.17%	1.43	0.32	1.75
HPV45-E7	TLQEIVLHL	7–15 (9)	0.24	99.96%	1.45	0.33	1.77
	NELDPVDLL	19–27 (9)	0.06	51.21%	1.44	0.36	1.80
	LQQLFLSTL	87–95 (9)	-0.06	37.63%	1.59	0.44	2.03
	QLFLSTLSF	89–97 (9)	-0.20	99.33%	1.30	1.14	2.44
	SSAEDLRTL	79–87 (9)	0.19	99.03%	1.56	0.39	1.95
	ELDPVDLLCY	20–29 (10)	0.01	57.92%	1.45	1.17	2.63
	LRTLQQLFL	84–92 (9)	-0.16	59.86%	1.80	0.43	2.23
	RETLQEIVL	5–13 (9)	0.12	46.68%	1.76	0.38	2.15
	LHLEPQNEL	13–21 (9)	0.03	74.50%	1.36	0.45	1.81
	QQLFLSTLSF	88–97 (10)	-0.13	37.17%	1.30	1.17	2.46

In the second step, five peptides out of ten selected peptides in the first step (Tables 6–8), which got better results in all analyses of both steps, were selected as the final-predicted epitopes. None of the final predicted epitopes showed more than 90% sequence similarity with mouse and human proteomes.

**Table 6:** The predicted epitopes from E5 oncoproteins in the second step selection.

Protein	Epitope	Docking			Conservancy	
		TDS	TDS	Inter-isoform	Inter-type*	Inter-hrHPV
		(HLA-I)	(HLA-II)	(% identity)	(% identity)	(100% identical)
HPV16-E5	FLIHTHARF	110	74	87.5	77.8	None
	SAFRCFIVY	111	73	79.2	55.6	None
	STYTSLIIL	131	62	16.7	44.4	None
	YTSLIILVL	112	67	16.7	33.3	None
	FLLCFCVLL	122	67	91.7	100.0	HPV16, 31 and 35
HPV18-E5	ATAFTVYVF	114	80	88.9	55.6	None
	CAYAWVLVF	135	70	100.0	77.8	None
	SPATAFTVY	117	61	88.9	77.8	None
	MCAYAWVLVF	130	71	100.0	70.0	None
	YAWVLVFVY	137	68	100.0	55.6	None
HPV31-E5	FVIHTHASF	143	71	14.3	77.8	None
	LSVSVYATL	149	67	100.0	66.7	None
	SVYATLLLL	139	72	100.0	44.4	None
	VVFIYIPLF	130	78	71.4	66.7	None
	VSVYATLLL	135	70	100.0	44.4	None
HPV45-E5	CAFAWLLVF	127	78	100.0	77.8	None
	YVCAFAWLL	109	62	100.0	55.6	None
	VYVCAFAWLL	127	56	100.0	60.0	None
	LHMHALHTL	111	82	66.7	55.6	None
	VITSPLTAF	125	69	100.0	88.9	None

*Between HPV16 and 31 (alpha- Papillomavirus 9), and between HPV18 and 45 (alpha- Papillomavirus 7)

**Table 7:** The predicted epitopes from E6 oncoproteins in the second step selection.

Protein	Epitope	Docking			Conservancy	
		TDS	TDS	Inter-isoform	Inter-type*	Inter-hrHPV
		(HLA-I)	(HLA-II)	(% identity)	(% identity)	(100% identical)
HPV16-E6	FAFRDLCIVY	110	66	96.6	80.0	None
	IVYRDGNPY	111	62	94.9	66.7	None
	CYSLYGTTL	131	73	63.6	77.8	None
	VYDFAFRDL	112	61	95.8	77.8	None
	KFYSKISEY	122	63	90.7	66.7	HPV16 and 35
HPV18-E6	KLPDLCTEL	114	62	87.5	100.0	HPV18 and 45
	TVLELTEVF	135	83	75.0	55.6	None
	FAFKDLFVVY	117	78	87.5	80.0	None
	LELTEVFEF	130	70	75.0	66.7	None
	DFYSRIREL	137	53	87.5	100.0	HPV18 and 45
HPV31-E6	FAFTDLTIVY	143	68	86.7	80.0	None
	RYSVYGTTL	149	70	100.0	77.8	None
	KVSEFRWYRY	139	51	100.0	60.0	None
	FRWYRYSVY	130	76	100.0	55.6	None
	YRYSVYGTTL	135	76	100.0	80.0	None
HPV45-E6	IVYRDCIAY	127	69	100.0	55.6	None
	KLPDLCTEL	109	65	56.3	100.0	HPV45 and 18
	YSRIRELRY	127	57	100.0	88.9	None
	ATLERTEVY	111	61	81.3	55.6	None
	FAFKDLCIVY	125	83	62.5	80.0	None

*Between HPV16 and 31 (alpha- Papillomavirus 9), and between HPV18 and 45 (alpha- Papillomavirus 7)

**Table 8:** The predicted epitopes from E7 oncoproteins in the second step selection.

Protein	Epitope	Docking			Conservancy	
		TDS	TDS	Inter-isoform	Inter-type*	Inter-hrHPV
		(HLA-I)	(HLA-II)	(% identity)	(% identity)	(100% identical)
HPV16-E7	RAHYNIVTF	120	61	100.0	66.7	None
	STHVDIRTL	107	57	76.5	77.8	None
	LEDLLMGTL	102	58	100.0	55.6	None
	LQPETTDLY	107	65	82.4	77.8	None
	TLHEYMLDL	90	55	94.1	66.7	None
HPV18-E7	SSADDLRAF	90	52	90.9	66.7	None
	FQQLFLNTL	105	67	45.5	77.8	None
	QLFLNTLSF	127	69	45.5	81.8	None
	TLQDIVLHL	94	59	90.9	88.9	None
	LRAFQQLFL	108	76	90.9	77.8	None
HPV31-E7	TSNYNIVTF	116	60	93.3	66.7	None
	TLQDYVLDL	111	64	100.0	66.7	HPV18 and 35
	GQAEPDTSNY	109	50	40.0	70.0	None
	QAEPDTSNY	102	59	40.0	66.7	None
	QPEATDLHCY	110	54	66.7	80.0	None
HPV45-E7	TLQEIVLHL	85	57	100.0	88.9	None
	NELDPVDLL	98	68	100.0	55.6	None
	LQQLFLSTL	100	60	100.0	77.8	None
	QLFLSTLSF	108	66	100.0	88.9	None
	SSAEDLRTL	84	55	26.7	66.7	None

*Between HPV16 and 31 (alpha- Papillomavirus 9), and between HPV18 and 45 (alpha- Papillomavirus

## Discussion

High prevalence and mortality of oncogenic infectious pathogens such as HPV and Helicobacter pylori have caused serious problems for humans. Currently, people who are infected with hrHPVs but show normal cytology or precancerous lesions do not have any treatment option, causing the disease progress toward invasive carcinoma in some cases. Unfortunately, no FDA-approved immunotherapy exists for pre-existing HPV infections or their related cancers to date. Immunotherapy of HPV-associated cancers by DNA or peptide-based vaccines, depends on the recognition of highly immunogenic epitopes, inducing robust and specific immune responses, particularly cell-mediated responses against the malignant cells.

The primary aim of this study was the prediction of CD8+ T cell epitope from the E5, E6 and E7 oncoproteins, using a comprehensive two-step selection plan. These proteins chose because they play a pivotal role in the cell transformation, immune evasion, and maintenance of malignancy, as well as, their permanent expression (E6 and E7) by the malignant cells [24–26]. Expression of E5 oncoprotein occurs in the early phase of HPV infection. Evidence indicates that E5 play a prominent role in the genesis of HPV-associated cancers, but is not essential for cancer progression [90], since when HPV genome integrates into the host genome, it usually results in the disruption of E1, E2, and E5 genes. Therefore, targeting E5 protein provides an opportunity for treatment of HPV infections and preventing the precancerous lesions from the progression to established carcinomas [20, 91]. Some genotypes of hrHPVs are more involved in the genesis of epithelial tissue malignancies [61]. Thus, in this study, hrHPV16, 18, 31 and 45 were targeted due to their high prevalence in the HPV-associated cancers, especially cervical carcinoma.

There are several limitations for epitope prediction: 1) The major drawback of peptide-based vaccines is low immunogenicity [92, 93]. Many studies have focused on enhancing immunogenicity using immune stimulating agents or adjuvants to avoid this problem. Another solution is the use of agonist epitopes [94]. Epitope immunogenicity is a crucial factor in vaccine development. However, many of known natural epitopes when are analyzed *in silico* by *IEDB MHC-I immunogenicity predictor*, do not obtain a high score. Therefore, in this study, epitope selection was based on the integrated approach, in which one analysis does not play an important role alone. 2) There are certain drawbacks associated with the function of each method invented for the MHC-peptide binding prediction [95]. For this reason, several predictors and a molecular docking program were used to augment the prediction accuracy. 3) Some web tools have been developed for MHC-II epitope prediction. Since MHC-II groove can bind to peptides with variable lengths, and different peptides have the different number of residues between their N-terminus and first anchor [54], the exact assignment of MHC-II core peptide would be a difficult problem which reduces the success rate of these prediction tools. Therefore, most MHC-II prediction tools did not usually make epitope predictions as accurately noted for MHC-I molecules [64, 96]. In cancer immunotherapy, the CTL-mediated responses play the central role in eradication of malignant cells, and the binding of epitopes to MHC-I molecules is an essential step for antigen presentation to CTLs. Thus, in this study, predicted epitopes were primarily selected by their MHC-I binding and processing scores. However, the MHC-II binding scores were actually of secondary importance to the epitope selection process as an extra advantage. Additionally, there are several other essential determinants which significantly affect the outcomes, such as antigen processing, immunogenicity, population coverage, conservancy and cross-reactivity with host antigens. Vaccine development requires a comprehensive approach to cover all these effectual elements, covered in this study.

The primary aim of molecular docking is the recognition of binding site of a ligand at a protein receptor surface, and docking and modeling the ligand into this recognized site. In this study, CABS-dock server was used for molecular docking analyses. CABS-dock has several main advantages: 1) The method does not require any data about the peptide structure and its binding site. 2) During docking process, peptide conformation is entirely flexible. 3) It is possible to apply dynamic conformational changes in the receptor structure and 4) to exclude some receptor regions from the docking search, leading to the more efficient search in the vicinity of the binding site at a sensible time. [88, 89].

In comparison with protein-ligand (small molecules) docking, Protein-peptide docking analysis is more problematic, since significant conformational changes occur during the process. As a general rule, how much the length of the query peptide to be longer, there are more torsions and conformational flexibilities. Additionally, in comparison to Protein-Protein interactions, Protein-peptide dockings are more transient, and their binding affinities are notably weaker [88]. These factors make structural predictions of long peptides very challenging. Therefore, in this study, 9mer peptides were preferred for selection compared to other possible lengths. They are also preferred by all MHC-I molecules as epitope and by MHC-II molecules as the core peptide of epitopes. Moreover, expansion of 9 or 10mer CTL epitopes to longer peptides may create a practical alternative, containing both CD4+ HTL and CD8+ CTL epitopes; Especially, when CD4+ HTL epitopes, covering CTL epitopes, are not recognized [97].

A large number of previous studies have used *in silico* analyses for epitope prediction against different pathogens [94, 96, 98–103]. However, the prediction of T cell epitopes inducing strong responses has remained a big challenge. For therapeutic HPV vaccines, many candidates have been designed to trigger the activation of CTLs or HTLs, mostly by targeting two major HPV oncoproteins, E6 or E7 [104], and in a few studies, E5 oncoprotein [98, 99]. As well as, several clinical trials have been launched for immunotherapy of HPV-associated cancers [46], although, they have not been so immunogenic, to induce a sufficient cellular immunity and eradicate malignant cells completely. Some studies have suggested that the use of E6 and E7 SLPs, containing both CD4+ HTL and CD8+ CTL epitopes, led to more potency and durability of CD8+ T cell reactivity *in vivo*, in comparison with the minimal CTL epitopes [97, 105].

In 1993, As pioneers in HPV epitope studies, Feltkamp *et al*. recognized the HPV16-E7 sequence RAHYNIVTF as an MHC-I epitope that can provoke CTL-mediated responses and eradicates established HPV l6-induced tumor cells in mice [106, 107]. This sequence is the first HPV16-E7 predicted epitope in our study as well.

In 2015, Kumar et al. studied HPV16-E5 oncoprotein to predict the candidate T-cell and B-cell epitopes [98]. They have screened 11 potent epitopes for MHC-I molecules according to PR and the immunogenicity score, using IEDB MHC-I binding and immunogenicity predictors. They found a 14mer potent epitope, SAFRCFIVYIIFVY, having the lowest PR and the highest immunogenicity score, *i*.*e*., 0.5 and 0.70, respectively. Notably, our second HPV16-E5 predicted epitope, SAFRCFIVY, is the N-terminal part of SAFRCFIVYIIFVY, and our first predicted epitope, FLIHTHARF, is the C-terminal part of the third epitope of their study, VYIPLFLIHTHARF.

In 2017, Tsang et al. scanned the HPV16-E6 and E7 oncoproteins for the match peptides with the consensus motif of HLA-A2 binding peptides [94]. The BIMAS algorithm [108] was employed to rank probable binding peptides according to the predicted *one-half-time dissociation* of pMHCs. Three potential CTL predicted epitopes of the E6 protein (KLPQLCTEL, KISEYRHYC, and QQYNKPLCDL) and three of the E7 protein (YMLDLQPET, TLHEYMLDL, and RTLEDLLMGT) were selected. They showed the immunogenicity of these peptides was enhanced when their agonist epitopes were used. The KLPQLCTEL and TLHEYMLDL sequences are the seventh and the fifth predicted epitopes of HPV16-E6 and HPV16-E7 in our study, respectively.

Experimental evidences about hrHPV-derived epitopes in literatures are mostly limited to E6 and E7 oncoproteins of HPV16 and 18. Among our first-step predicted epitopes: FLLCFCVLL and YIIFVYIPL from the E5-derived epitopes [109], FAFRDLCIVY [110], CYSLYGTTL [111], VYDFAFRDL [111, 112], KFYSKISEY [113], KLPQLCTEL [114–116], ISEYRHYCY [117], EYRHYCYSL [111], KLPDLCTEL [116, 118–120], FAFKDLFVV [119, 120] and KLPDLCTEL [116, 118–120] from the E6-derived epitopes, RAHYNIVTF [121], LEDLLMGTL [122], TLHEYMLDL [115, 122–124], LLMGTLGIV [115, 116, 125, 126], QAEPDRAHY [117], GTLGIVCPI [115, 126], FQQLFLNTL [127] and TLQDIVLHL [119] from the E7-drived epitopes were reported as T-cell epitopes experimentally. Besides, IVYRDGNPY, CYSLYGTTL, KLPQLCTEL and ISEYRHYCY from the E6-derived epitopes, and RAHYNIVTF and GTLGIVCPI from the E7-derived epitopes were also reported as HLA ligands [128]. Others are novel epitopes that they also require experimental studies for validation.

As far as we know, this is the first time that in a laborious *in silico* study for epitope prediction, E5, E6 and E7 oncoproteins of hrHPV16, 18, 31 and 45 have been investigated altogether. Moreover, in previous studies, usually only one predictor tool was used for making epitope prediction, or if several tools were used, no integrated approach was employed to make the conclusion. We believed that our predicted epitopes are valuable candidates for further *in vitro* and *in vivo* therapeutic vaccine studies. Additionally, the introduction of the ten epitopes for each HPV genotype oncoprotein in the first step of the study shows which region of each oncoprotein is rich of the epitope, and thus, is more suitable for use in the design of SLPs. Notably, the previous *in vivo* studies have been conducted using SLPs of hrHPV-E6 and/or–E7 oncoproteins, in particular HPV16 oncoproteins [92, 129–133]. Furthermore, the two-step plan of this *in silico* study, which each step includes several analyses to find proper epitopes by an integrated approach, would provide a basis for rational epitope prediction. However, it could be more efficient by adding other useful analyses. Further studies are recommended on the peptide binding assays, the design of polyepitope constructions including E5, E6 and E7 epitopes, the expansion of the minimal CTL epitopes to longer peptides (SLPs), the use of various adjuvants, involvement of delivery routes, mouse immunization with the designed constructs, evaluation of immune responses such as cytokines, antibodies, CTLs and tumor growth for finding the best construct for clinical trials. It is important that improper vaccine design and immunosuppressive microenvironment were known as the main reasons of the failure in cancer immunotherapy by therapeutic cancer vaccines [134].

## Supporting information

S1 File HPV16, 18, 31 and 45 oncoprotein sequences. (ZIP)

S2 File CABS-dock procedure. (ZIP)

S3 File MHC-I and II binding predictions. (ZIP)

S4 File MHC-I immunogenicity predictions. (ZIP)

S5 File MHC-I population coverages predictions. (ZIP)

S6 File MHC-I and II molecular docking analyses. (ZIP)

S7 File Epitope conservation analyses. (ZIP)

S1 Table MHC-I binding prediction alleles. (ZIP)

S2 Table Syfpeithi MHC-I binding prediction reference sequences. (ZIP)

S3 Table MHC II binding predictions alleles. (ZIP)
