# Supplementary material for: A comprehensive in silico analysis for identification of therapeutic epitopes in HPV16, 18, 31 and 45 oncoproteins
Source: PLoS One. 2018 Oct 24;13(10):e0205933. doi: 10.1371/journal.pone.0205933 (PMC6200245; doi:10.1371/journal.pone.0205933)
Supplement: S2 File — (ZIP) [file pone.0205933.s002.zip › S2 File (CABS-dock procedure)/CABS-dock procedure .docx]

# CABS-dock procedure

The fully automated CABS-dock procedure contains four following main stages:

## Stage 1: flexible docking with the CABS algorithm

CABS-dock gets the 3D structure of the receptor and the sequence of the peptide as obligatory inputs. Also, there are some non-obligatory inputs as recommendations which could improve outputs [[1](#_ENREF_1)].

CABS-dock executes a flexible docking by the CABS algorithm and makes 10,000 models (protein-peptide complex), sorted in 10 trajectories. Each trajectory contains 1000 models. During docking, CABS-dock lets full flexibility of the peptide and small fluctuations of the protein backbone. In stage 1 and 2, It constructs only the C-alpha coordinates of the residues in the PDB format.

## Stage 2: initial model filtering

In this stage, all unbound states (that cause protein-peptide interaction energy to be equal to zero), are excluded from the analysis, and up to 1000 probable correct models (100 models from each trajectory) which hold the lowest interaction energy are chosen for the next stage. This approach usually keeps the best of the simulated models in the remained set [[2](#_ENREF_2)].

## Stage 3: structural clustering of models

The 1000 reminded models are sorted into 10 clusters by the k-medoid clustering protocol. The clustering is run 100 times with different initial seeds and k = 10. Consensus medoids (a representative model of a cluster whose average dissimilarity to all the elements in the cluster is minimal) are selected as the final models. Eventually, the cluster density is used to rank the final representative models. Cluster density is equal to the number of elements (CD) in a cluster divided by their average ligand RMSD (root mean square deviation). The higher the CD value, the greater the accuracy. Ligand RMSD value shows the differentiation measure between cluster elements. As a guideline, RMSD < 3.0 A ̊ means high accuracy, RMSD ≥ 3.0 and ≤ 5.5 A ̊ means medium accuracy, and RMSD > 5.5 A ̊ means low accuracy [[2](#_ENREF_2)].

## Stage 4: Reconstruction and local optimization of the final models

The last stage of the CABS-dock procedure is the combined reconstruction and local optimization of the final models (10 medoids). The reconstruction of the final models (10 medoids) from C-alpha trace to all-atom models is done by using the MODELLER [[3](#_ENREF_3)] program. A two-step procedure treats the reconstruction of atomic details. In the first step, just the backbone atoms are reconstructed. In the next step, all atoms of the side chains are added to the backbone, and then, the model is optimized to eliminate structural errors, such as incorrect bond angles and lengths, or steric clashes [[2](#_ENREF_2)].

The optimization is performed by the minimization of the DOPE (Discreet Optimized Protein Energy) potential [[4](#_ENREF_4)]. As a consequence, energy minimized all-atom representations of the final models are made, and the all-atom coordinates are formulated in the PDB format[[2](#_ENREF_2)].

In the result page, the PDB file, CD, average RMSD, maximum RMSD and number of cluster elements, of top-ranked models are provided as final outputs. The PDB file could be downloaded for the visualization or further analysis. Also, the 'contact maps' tab allows studying the interactions between the peptide and the protein.

References

1. Kurcinski M, Jamroz M, Blaszczyk M, Kolinski A, Kmiecik S. CABS-dock web server for the flexible docking of peptides to proteins without prior knowledge of the binding site. Nucleic Acids Research. 2015;43(W1):W419-W24.

2. Blaszczyk M, Kurcinski M, Kouza M, Wieteska L, Debinski A, Kolinski A, et al. Modeling of protein–peptide interactions using the CABS-dock web server for binding site search and flexible docking. Methods. 2016;93:72-83.

3. Benjamin W, Sali A. Comparative protein structure modeling using. Modeller Curr Protoc bioinfo. 2014;47:5-6.

4. Shen My, Sali A. Statistical potential for assessment and prediction of protein structures. Protein science. 2006;15(11):2507-24.
