# Supplementary material for: A comprehensive in silico analysis for identification of therapeutic epitopes in HPV16, 18, 31 and 45 oncoproteins
Source: PLoS One. 2018 Oct 24;13(10):e0205933. doi: 10.1371/journal.pone.0205933 (PMC6200245; doi:10.1371/journal.pone.0205933)
Supplement: S1 Table — (ZIP) [file pone.0205933.s008.zip › S1 Table (MHC-I binding prediction alleles)/MHC-I binding prediction alleles.docx]

.

Table S1. Human and mouse alleles used for MHC-I binding predictions.

| Species | server | MHC I alleles |
| --- | --- | --- |
| Human | IEDB  NetMHCpan4 | HLA-A*01:01, HLA-A*02:01, HLA-A*02:06, HLA-A*03:01, HLA-A*11:01, HLA-A*23:01,  HLA-A*24:02, HLA-A*25:01, HLA-A*26:01, HLA-A*29:02, HLA-A*30:01, HLA-A*30:02,  HLA-A*31:01, HLA-A*32:01, HLA-A*33:03, HLA-A*68:01, HLA-A*68:02, HLA-A*74:01,  HLA-B*07:02, HLA-B*08:01, HLA-B*13:01, HLA-B*13:02, HLA-B*14:02, HLA-B*15:01,  HLA-B*15:02, HLA-B*15:25, HLA-B*18:01, HLA-B*27:02, HLA-B*27:05, HLA-B*35:01,  HLA-B*35:03, HLA-B*37:01, HLA-B*38:01, HLA-B*39:01, HLA-B*40:01, HLA-B*40:02,  HLA-B*44:02, HLA-B*44:03, HLA-B*46:01, HLA-B*48:01, HLA-B*49:01, HLA-B*50:01,  HLA-B*51:01, HLA-B*52:01, HLA-B*53:01, HLA-B*55:01, HLA-B*56:01, HLA-B*57:01,  HLA-B*58:01, HLA-B*58:02, HLA-C*01:02, HLA-C*02:02, HLA-C*02:09, HLA-C*03:02,  HLA-C*03:03, HLA-C*03:04, HLA-C*04:01, HLA-C*05:01, HLA-C*06:02, HLA-C*07:01,  HLA-C*07:02, HLA-C*07:04, HLA-C*08:01, HLA-C*08:02, HLA-C*12:02, HLA-C*12:03,  HLA-C*14:02, HLA-C*15:02, HLA-C*16:01, HLA-C*17:01, HLA-E*01:01, HLA-E*01:03,  HLA-G*01:02, HLA-G*01:03, HLA-G*01:04 and HLA-G*01:06 |
|  | Rankpep | HLA-A*01:01 T cell epitope (9mer), HLA-A*01:01 All (9mer), HLA-A*02:01 (EPI-OPT),  HLA-A*02:01 (HIGH BINDERS), HLA-A*02:01 (8, 9, 10 and 11mer), HLA-A*02:06 (9mer), HLA-A*03:01 (9, 10 and 11mer), HLA-A*11:01 (9 and 10mer), HLA-A*24:02 (9, 10 and 11mer), HLA-A*29:02 (9mer), HLA-A*31:01 (9 and 10mer), HLA-A*68:01 (9, 10 and 11mer), HLA-A*68:02 (9mer), HLA-B*07:02 (9mer), HLA-B*08 (8, 9, 10 and 11mer), HLA-B*14 9mer), HLA-B*15:01 (9 and 10mer), HLA-B*15:02 (9mer), HLA-B*18 (9mer), HLA-B*27:02 (9mer), HLA-B*27:05 (8, 9 and 10mer), HLA-B*35:01 (8, 9, 10, 11mer), HLA-B*38:01 (9mer), HLA-B*39:01 (8 and 9mer), HLA-B*44:02 (9mer), HLA-B*44:03 (9mer), HLA-B*51:01 (9mer), HLA-B*53:01 (9 and 10mer), HLA-B*57:01 (9mer), HLA-B*58:01 (9mer), HLA-C*01:02 (9mer), HLA-C*03:04 (9mer), HLA-C*07:02 (9mer), HLA-E (9mer), HLA-G (9mer) |
|  | SYFPEITHI | HLA-A*01, HLA-A*02:01, HLA-A*03, HLA-A*11:01, HLA-A*24:02, HLA-A*26, HLA-A*68:01, HLA-B*07:02, HLA-B*08, HLA-B*13, HLA-B*14:02, HLA-B*15:01, HLA-B*18, HLA-B*27:05, HLA-B*35:01, HLA-B*37, HLA-B*38:01, HLA-B*39:01, HLA-B*40:01, HLA-B*44:02,  HLA-B*49:01, HLA-B*50:01, HLA-B*51:01, HLA-B*53:01, HLA-B*57:01, HLA-B*58:02 |
| Mouse | IEDB  NetMHCpan3  Rankpep  SYFPEITHI | H-2-Db, H-2-Dd, H-2-Kb, H-2-Kd, H-2-Kk and H-2-Ld  H-2-Db, H-2-Dd, H-2-Kb, H-2-Kd, H-2-Kk, H-2-Ld, H-2-Qa1 and H-2-Qa2  H-2-Db (8, 9, 10 and 11mer), H-2-Dd (9, 10 and 11mer), H-2-Kb (8 and 9mer), H-2-KD (8, 9, 10 and 11mer), H-2-Kk (8 and 9mer), H-2-Ld(9mer), H-2-Qa2a  H-2-Db, H-2-Kb, H-2-Kd, H-2-Kk and H-2-Ld |
