# Supplementary material for: A comprehensive in silico analysis for identification of therapeutic epitopes in HPV16, 18, 31 and 45 oncoproteins
Source: PLoS One. 2018 Oct 24;13(10):e0205933. doi: 10.1371/journal.pone.0205933 (PMC6200245; doi:10.1371/journal.pone.0205933)
Supplement: S2 Table — (ZIP) [file pone.0205933.s009.zip › S2 Table (Syfpeithi MHC-I binding prediction reference sequences)/Syfpeithi MHC-I binding prediction reference sequences.docx]

Table S2. Reference sequences used in the Syfpeithi MHC-I binding predictions.

| Allele/Allele group | Reference sequence | Sequence source | Reference score |
| --- | --- | --- | --- |
| HLA-A*01 | YTDPGFWYY | Rankpep | 36 |
| HLA-A*02:01 | LLIRPLLLSV | HPV 16-E5 | 31 |
| HLA-A*03 | IVCPICSQK | HPV 16-E7 | 31 |
| HLA-A*11:01 | SVYGDTLEK | HPV 18-E6 | 29 |
| HLA-A*24:02 | VYGETLEKI | HPV 45-E6 | 27 |
| HLA-A*26 | EVYDFAFRDL | HPV 16-E6 | 29 |
| HLA-A*68:01 | TTLEKLTNK | HPV 31-E6 | 25 |
| HLA-B*07:02 | KPLNPAEKL | HPV 18-E6 | 24 |
| HLA-B*08 | HLKWKWWTL | Rankpep | 37 |
| HLA-B*13 | CQCKSTLRL | HPV 31-E7 | 24 |
| HLA-B*14:02 | LRTLQQLFL | HPV 45-E7 | 30 |
| HLA-B*15:01 | GQGPFGTQY | Rankpep | 23 |
| HLA-B*18:01 | DELRLNCVY | HPV 31-E6 | 28 |
| HLA-B*27:05 | RRYWGLIHR | Rankpep | 30 |
| HLA-B*35:01 | QPEATDLHCY | HPV 31-E7 | 23 |
| HLA-B*37 | AEPQRHTML | HPV 18-E7 | 28 |
| HLA-B*38:01 | YHDEAHSYF | Rankpep | 22 |
| HLA-B*39:01 | LHIHAILSL | HPV 18-E5 | 27 |
| HLA-B*40:01 | RETLQEIVL | HPV 45-E7 | 28 |
| HLA-B*44:02 | AEPQRHKIL | HPV 45-E7 | 27 |
| HLA-B*49:01 | LEIPYDELRL | HPV 31-E6 | 26 |
| HLA-B*50:01 | TETEVLDFA | HPV 31-E6 | 21 |
| HLA-B*51:01 | DPQERPRKL | HPV 16-E6 | 26 |
| HLA-B*53:01 | FPWCPFHWF | Rankpep | 23 |
| HLA-B*57:01 | ITPPHQDAW | Rankpep | 20 |
| HLA-B*58:02 | FAFRDLCI | HPV 16-E6 | 28 |
| H-2-Db | FCIHNCDYM | Rankpep | 28 |
| H-2-Kb | DLYCYEQL | HPV 16-E7 | 28 |
| H-2-Kd | RYSVYGTTL | HPV 31-E6 | 28 |
| H-2-Kk | TELYNLLI | HPV 45-E6 | 25 |
| H-2-Ld | YPHPGPHYF | Rankpep | 28 |
