# Supplementary material for: A comprehensive in silico analysis for identification of therapeutic epitopes in HPV16, 18, 31 and 45 oncoproteins
Source: PLoS One. 2018 Oct 24;13(10):e0205933. doi: 10.1371/journal.pone.0205933 (PMC6200245; doi:10.1371/journal.pone.0205933)
Supplement: S2 Table — (ZIP) [file pone.0205933.s009.zip › S2 Table (Syfpeithi MHC-I binding prediction reference sequences)/Syfpeithi MHC-I binding prediction reference sequences.pdf]

Table S2. Reference sequences used in the Syfpeithi MHC-I binding predictions.

Allele/Allele group	Reference sequence	Sequence source	Reference score
HLA-A*01	YTDPGFWYY	Rankpep	36
HLA-A*02:01	LLIRPLLLSV	HPV 16-E5	31
HLA-A*03	IVCPICSQK	HPV 16-E7	31
HLA-A*11:01	SVYGDTLEK	HPV 18-E6	29
HLA-A*24:02	VYGETLEKI	HPV 45-E6	27
HLA-A*26	EVYDFAFRDL	HPV 16-E6	29
HLA-A*68:01	TTLEKLTNK	HPV 31-E6	25
HLA-B*07:02	KPLNPAEKL	HPV 18-E6	24
HLA-B*08	HLKWKWWTL	Rankpep	37
HLA-B*13	CQCKSTLRL	HPV 31-E7	24
HLA-B*14:02	LRTLQQLFL	HPV 45-E7	30
HLA-B*15:01	GQGPFGTQY	Rankpep	23
HLA-B*18:01	DELRLNCVY	HPV 31-E6	28
HLA-B*27:05	RRYWGLIHR	Rankpep	30
HLA-B*35:01	QPEATDLHCY	HPV 31-E7	23
HLA-B*37	AEPQRHTML	HPV 18-E7	28
HLA-B*38:01	YHDEAHSYF	Rankpep	22
HLA-B*39:01	LHIHAILSL	HPV 18-E5	27
HLA-B*40:01	RETLQEIVL	HPV 45-E7	28
HLA-B*44:02	AEPQRHKIL	HPV 45-E7	27
HLA-B*49:01	LEIPYDELRL	HPV 31-E6	26
HLA-B*50:01	TETEVLDFA	HPV 31-E6	21
HLA-B*51:01	DPQERPRKL	HPV 16-E6	26
HLA-B*53:01	FPWCPFHWF	Rankpep	23
HLA-B*57:01	ITPPHQDAW	Rankpep	20
HLA-B*58:02	FAFRDLCI	HPV 16-E6	28
H-2-Db	FCIHNCDYM	Rankpep	28
H-2-Kb	DLYCYEQL	HPV 16-E7	28
H-2-Kd	RYSVYGTTL	HPV 31-E6	28
H-2-Kk	TELYNLLI	HPV 45-E6	25
H-2-Ld	YPHPGPHYF	Rankpep	28
