# Supplementary material for: A comprehensive in silico analysis for identification of therapeutic epitopes in HPV16, 18, 31 and 45 oncoproteins
Source: PLoS One. 2018 Oct 24;13(10):e0205933. doi: 10.1371/journal.pone.0205933 (PMC6200245; doi:10.1371/journal.pone.0205933)
Supplement: S3 Table — (ZIP) [file pone.0205933.s010.zip › S3 Table (MHC II binding predictions alleles)/MHC II binding prediction alleles.docx]

Table S3. Human and mouse alleles used for MHC-II binding prediction.

| Species | Server | MHC I alleles |
| --- | --- | --- |
| Human | IEDB | HLA-DRB1*01:01, HLA-DRB1*03:01 , HLA-DRB1*04:01, HLA-DRB1*04:05, HLA-DRB1*07:01,  HLA-DRB1*08:02, HLA-DRB1*09:01 , HLA-DRB1*11:01, HLA-DRB1*12:01, HLA-DRB1*13:02,  HLA-DRB1*15:01, HLA-DRB3*01:01 , HLA-DRB3*02:02, HLA-DRB4*01:01, HLA-DRB5*01:01,  HLA-DQA1*05:01/DQB1*02:01, HLA-DQA1*05:01/DQB1*03:01,  HLA-QA1*03:01/DQB1*03:02, HLA-DQA1*04:01/DQB1*04:02 ,  HLA-DQA1*01:01/DQB1*05:01, HLA-DQA1*01:02/DQB1*06:02,  HLA-DPA1*02:01/DPB1*01:01, HLA-DPA1*01:03/DPB1*02:01, HLA-DPA1*01/DPB1*04:01,  HLA-DPA1*03:01/DPB1*04:02, HLA-DPA1*02:01/DPB1*05:01,  HLA-DPA1*02:01/DPB1*14:01 |
| Mouse | IEDB | H-2-IAb, H-2-IAd and H-2-IEd |
